# Life without a brain: Neuroradiological and behavioral evidence of neuroplasticity necessary to sustain brain function in the face of severe hydrocephalus

**DOI:** 10.1038/s41598-019-53042-3

**Published:** 2019-11-11

**Authors:** C. F. Ferris, X. Cai, J. Qiao, B. Switzer, J. Baun, T. Morrison, S. Iriah, D. Madularu, K. W. Sinkevicius, P. Kulkarni

**Affiliations:** 1 Center for Translational NeuroImaging, Northeastern University, Boston, MA USA; 2 NeuroScience Associates, Knoxville, TN USA; 3 Alexion Pharmaceuticals, New Heaven, CT USA

**Keywords:** Disease model, Hydrocephalus

## Abstract

A two-year old rat, R222, survived a life-time of extreme hydrocephaly affecting the size and organization of its brain. Much of the cortex was severely thinned and replaced by cerebrospinal fluid, yet R222 had normal motor function, could hear, see, smell, and respond to tactile stimulation. The hippocampus was malformed and compressed into the lower hindbrain together with the hypothalamus midbrain and pons, yet R222 showed normal spatial memory as compared to age-matched controls. BOLD MRI was used to study the reorganization of R222’s brain function showing global activation to visual, olfactory and tactile stimulation, particularly in the brainstem/cerebellum. The results are discussed in the context of neuroadaptation in the face of severe hydrocephaly and subsequent tissue loss, with an emphasis on what is the “bare minimum” for survival.

## Introduction

In an era where brain imaging is common place around the world, more and more cases of extreme alterations in brain morphology appear in the literature^1,2^ and popular press. Usually caused by early hydrocephalus, large regions of the brain can be reduced and malformed. Historically, there have been numerous medical reports of severe hydrocephalus with debilitating cognitive and motor problems and shortened life spans. However, in some rare cases, the affected individuals can be highly intelligent with no obvious physical abnormalities or sensory motor deficits^3^. John Lorber, a British neurologist studied one such patient and following imaging reported “… the boy has virtually no brain”^4^. The cortical mantle, in this and many other cases of severe hydrocephalus, can be reduced to a ribbon of tissue. The localization of function to specific areas of the central nervous system is a fundamental axiom of developmental neurobiology across the mammalian kingdom. However, when forced to redraft nature’s plan due to severe limitations in space, as in many human cases, or rat R222 described here, how does the brain reorganize itself?

R222 was discovered by chance while imaging a cohort of two years-old rats. Much of the brain was deformed and unrecognizable, replaced by cerebrospinal fluid; yet, R222’s general health, appearance and body weigh were no different from the other rats in the cohort. There is a vast literature on the neurobiological and behavioral effects of hydrocephaly in children and adults^5^. Without medical intervention to reduce the expansion of the ventricular space these individuals present with various cognitive and motor disorders and shortened life spans. Experimental models creating hydrocephaly in new borne and adult animals report the rapid development of neuropathology, particularly in white matter tracts around the expanding ventricles, and a constellation of sensory and motor deficits comorbid with failing health^5^. What made R222 unique with respect to human examples of severe hydrocephalus and experimental models of hydrocephaly in animals was its age - by rat standards R222 was old, comparable in age to a 70 year old human^6^. Living to old age with untreated hydrocephalus is very rare. We immediately recognized the opportunity to use magnetic resonance imaging (MRI) to interrogate the brain’s functional capabilities and characterize the reorganization of gray and white matter. To this end, BOLD (blood oxygen level dependent) imaging was used to assess brain activity under resting conditions and in response to olfactory, visual and tactile stimulation. In addition, R222 was also tested for cognitive and motor functions and, in the end, the brain was harvested for immunohistochemical analysis of the acetylcholine, dopamine and norepinephrine.

## Methods

### Image acquisition

The high resolution neuroanatomy and BOLD imaging in response to provocation paradigms involving odor and foot shock have been described previously^7–9^. In brief, rats were scanned using a quadrature transmit/receive volume coil built into the rat head holder and restraining system for animal imaging at 300 MHz (Animal Imaging Research, Holden, MA USA). A Bruker Biospec 7.0 T/20-cm USR horizontal magnet was used to conduct all experiments (Bruker, Billerica, Massachusetts) with a 20-G/cm magnetic field gradient insert (ID = 12 cm) capable of a 120-µs rise time (Bruker). High-resolution anatomical data sets were collected for all rats using a RARE pulse sequence (22 slice; 1.0 mm; field of vision [FOV] 3.0 cm; 256 × 256; repetition time [TR] 2.5 sec; echo time [TE] 12msec; NEX 2; 2 min acquisition time. Functional images for R222 were acquired using spin echo (SE) BOLD, a multi-slice HASTE pulse sequence (Half Fourier Acquisition Single Shot Turbo Spin Echo). Each scanning session was continuous, starting with baseline image acquisitions, followed by a single stimulus (almond odor) or repetitive on/off stimuli for vision and touch. Using voxel-based analysis, the percent change in BOLD signal for each independent voxel was averaged and compared to baseline.

### Resting state functional connectivity

Resting state BOLD functional connectivity used in this study has been described previously^10^. In brief, preprocessing combined Analysis of Functional NeuroImages (AFNI_17.1.12, http://afni.nimh.nih.gov/afni/), FMRIB Software library (FSL, v5.0.9, http://fsl.fmrib.ox.ac.uk/fsl/), Deformable Registration via Attribute Matching and Mutual-Saliency Weighting (DRAMMS 1.4.1, https://www.cbica.upenn.edu/sbia/software/dramms/index.html) and MATLAB (Mathworks, Natick, MA). Brain tissue masks were manually drawn using 3DSlicer (https://www.slicer.org/) and applied for skull-stripping. Motion outliers (i.e., data corrupted by extensive motion) were detected and regressed out. Motion spikes were identified and removed from the time-course signals. This filtering step was followed by slice timing correction from interleaved slice acquisition order. Band-pass filtering (0.01 Hz~0.1 Hz) was preformed to reduce low-frequency drift effects and high-frequency physiological noise. The resulting images were further detrended and spatially smoothed (full width at half maximum = 0.8 mm). Finally, regressors comprised of motion outliers, the six motion parameters, the mean white matter, and cerebrospinal fluid time series were fed into general linear models for nuisance regression to remove unwanted effects. The region-to-region functional connectivity method was performed to measure correlations in spontaneous BOLD fluctuations with the olfactory bulb. Voxel time series data were averaged based on the residual images using the nuisance regression procedure with motion parameters and mean time courses of white matter and ventricles. Pearson’s correlation coefficients were computed to assess the interregional temporal correlations. The r-values (ranging from −1 to 1) were z-transformed using the Fisher’s Z transform to improve normality.

### Neurohistology embedding, sectioning and staining

Brains were examined, then treated overnight with 20% glycerol and 2% dimethylsulfoxide to prevent freeze-artifacts. The specimens were then embedded in a gelatin matrix using MultiBrainTechnology (NeuroScience Associates, Knoxville, TN). The blocks were rapidly frozen, after curing by immersion in 2-Methylbutane chilled with crushed dry ice and mounted on a freezing stage of an AO 860 sliding microtome. The MultiBrain® blocks were sectioned in coronally on the microtome. All sections were cut through the entire length of the specimen segment and collected sequentially into series of 24 containers. All containers contained Antigen Preserve solution (50% PBS pH 7.0, 50% Ethylene Glycol, 1% Polyvinyl Pyrrolidone); no sections were discarded.

For the Thionine Nissl Stain, set of every twelfth section was mounted on gelatin coated glass slides, air dried and carried through the following sequence: 95% ethanol, 95% ethanol/Formaldehyde; 95% ethanol, Chloroform/Ether/absolute ethanol (8:1:1), 95% ethanol; 10% HCl/ethanol, 95% ethanol, 70% ethanol, deionized water, Thionine (0.05% Thionine/acetate buffer, pH 4.5), deionized water, 70% ethanol, 95% ethanol, Acetic Acid/ethanol, 95% ethanol, 100% ethanol, 100% ethanol, 1:1 100% ethanol/xylene, xylene, xylene, coverslip.

For CFN-1-2 immunochemistry, every twelfth section at an interval of 480 µm was stained free-floating. All incubation solutions from the primary antibody onward used Tris buffered saline (TBS) with Triton X100 as the vehicle; all rinses were with TBS. After hydrogen peroxide treatment, the sections were immunostained with the primary antibodies (NeuN IHC), overnight at room temperature. Vehicle solutions contained TritonX-100 for permeabilization. Following rinses, a biotinylated secondary antibody (anti IgG of host animal in which the primary antibody was produced) was applied. After further rinses Vector Lab’s ABC solution (avidin-biotin-HRP complex; details in instruction for VECTASTAIN® Elite ABC, Vector, Burlingame, CA) was applied. The sections were again rinsed, then treated with diaminobenzidine tetrahydrochloride (DAB) and hydrogen peroxide to create a visible reaction product. Following further rinses, the sections were mounted on gelatin coated glass slides, then air dried. The slides were dehydrated in alcohols, cleared in xylene and coverslipped.

For CFN-1immunochemistry, every twelfth section at an interval of 360 µm was stained free-floating. All incubation solutions from the primary antibody onward used TBS with Triton X100 as the vehicle; all rinses were with TBS. After a hydrogen peroxide treatment the sections were immunostained with the primary antibodies (Tyrosine Hydroxylase IHC, Serotonin 5HT, ChAT IHC, MBP (SMI-99) IHC) overnight at room temperature. Vehicle solutions contained TritonX-100 for permeabilization. Following rinses, a biotinylated secondary antibody (anti IgG of host animal in which the primary antibody was produced) was applied. After further rinses Vector Lab’s ABC solution (avidin-biotin-HRP complex; details in instruction for VECTASTAIN® Elite ABC, Vector, Burlingame, CA) was applied. The sections were again rinsed, then treated with DAB and hydrogen peroxide to create a visible reaction product. Following further rinses, the sections were mounted on gelatin coated glass slides, then air dried. The slides were dehydrated in alcohols, cleared in xylene and cover slipped. Details for primary antibodies (i.e. catalog number, manufacturer etc.) can be found in Supplementary Material.

Each slide was laser etched with the block number and the stain. Following serial ordering of the slides, rostral to caudal for each stain, the slides were numbered by permanent ink in the upper right corner.

### Behavioral testing

The novel object preference task (NOP) was used to assess episodic learning and memory as previously described^11^. The apparatus consisted of a black cube-shaped Plexiglass box (L:60.9 W: 69.2 H:70.5 cm) with no lid, indirectly illuminated with two 40 W incandescent bulbs. Animals were placed in the empty box (15 min) for acclimation on day one. On day two, for the familiar phase (5 min), animals were placed in the box with two identical objects arranged in diagonal corners, 5 cm from each wall. After a 90 min rest period in their home cage, animals were placed back in the box for the novel phase (3 min) with one of the familiar objects and a novel object.

The Barnes Maze was used to assess spatial learning and memory^11^. The maze consists of a circular platform (121 cm in diameter, elevated 40 cm), with 18 escape holes along the perimeter at 30 cm intervals. A black, removable enclosed Plexiglas goal box was positioned under a single escape hole on the underside of the maze (L:40.0 × W:12.7 × H:7.6 cm) in the same position relative to the testing room across all trials. Between trials, the maze was rotated 45 degrees and the goal box shifted accordingly for cardinal consistency. Animals were placed inside the goal box for 1 min and then under an enclosed container at the center of the circular platform for 30 s, that was then lifted to start the trial. If animals did not find the goal box within the test period (4 min), they were gently nudged into the box and allowed to stay for 1 min, and then placed back in their home cages between trials (3 trials/day for 4 days). For both the NOR and the Barnes maze, all trials were video recorded and analyzed using manual methods and verified with automated scoring using ANY-maze® software (Stoelting, Wood Dale, IL).

A tapered balance beam (Dragonfly Inc., Ridgeley, WV) and rota-rod were used to measure motor behavior^10^. The balance beam (L: 150 cm, W: 5.5 cm tapering down to 1.5 cm, elevated 120 cm) was equally divided into three sections (L:47 cm each; “wide”, “middle”, “thin” sections) that were lined with touch-sensitive sensor ledges (Width: 2 cm) that ran the length of the beam and were arranged on each side, 4 cm below the surface of the beam to count paw slips (or *foot faults*). At the start of the maze (“wide” section), was a wooden start platform, and at the end of the beam (immediately following the “thin” section) was a black enclosed Plexiglas goal box. After 2 days of training (3 trials per day), animals were tested (3 trials/day for 2 days). Prior to each trial, animals were placed inside the goal box for 1 min. Animals were then placed on a start platform and timed for traversing into the goal box, where they remained for 1 min, and were then placed back in their home cage until the next trial (30 min intertrial interval).

Following 2 days of training (3 trials/day), animals were tested over 2 days (3 trials/day) using the rota-rod by placing them on a rotating cylinder (diameter: 4 cm) that rotated at an increasing frequency starting at 1 rpm and increasing linearly at a 0.1 v/t2 acceleration rate for a total of 210 seconds ending at a max frequency of 50 rpm^10^. Latency to fall off the rod was recorded for each animal and averaged across trials and days. For all behavioral measures, GraphPad Prism version 6.0 (GraphPad Software, La Jolla, CA) was used for statistical analyses. One-sample t-tests assessed differences from chance levels (i.e., =50%) of exploration in the NOR task, for each experimental group individually.

Rats were housed in Plexiglas cages (two per cage) and maintained in ambient temperature (22–24 °C) on a 12:12 light:dark cycle (lights on at 07:00 a.m.). Food and water were provided ad libitum. Rats were imaged during the light phase of the circadian cycle. All rats were acquired and cared for in accordance with the guidelines published in the NIH Guide for the Care and Use of Laboratory Animals. All methods and procedures described below were pre-approved by the Northeastern University Institutional Animal Care and Use Committee.

## Results

### Neuroanatomy

**Figure 1:**
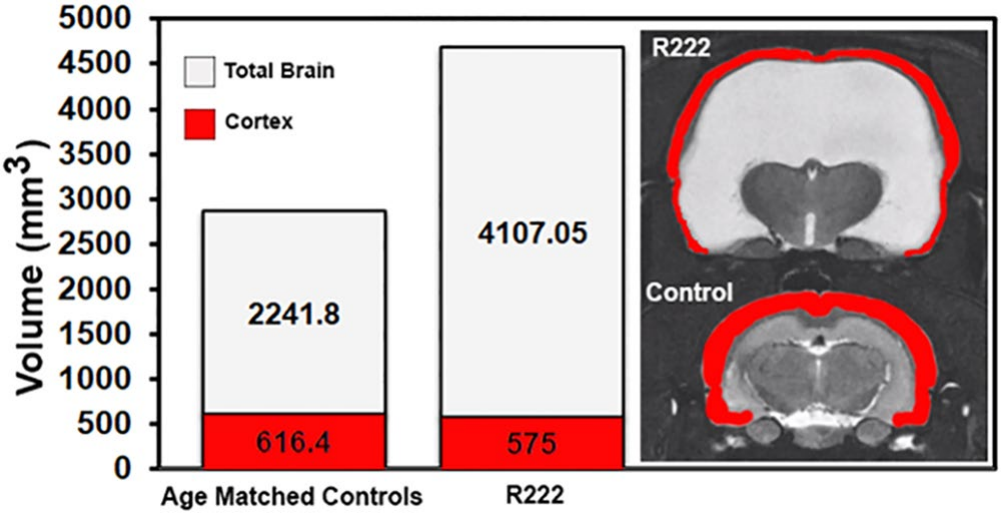
Brain and Cortical Volumes. Shown is the average total brain volume (brain and CSF filled ventricles) of five age matched controls and R222. The MR images depict the actual size of R222’s brain as compared to that of an age matched control with the cortices in each highlighted in red.

**Figure 2:**
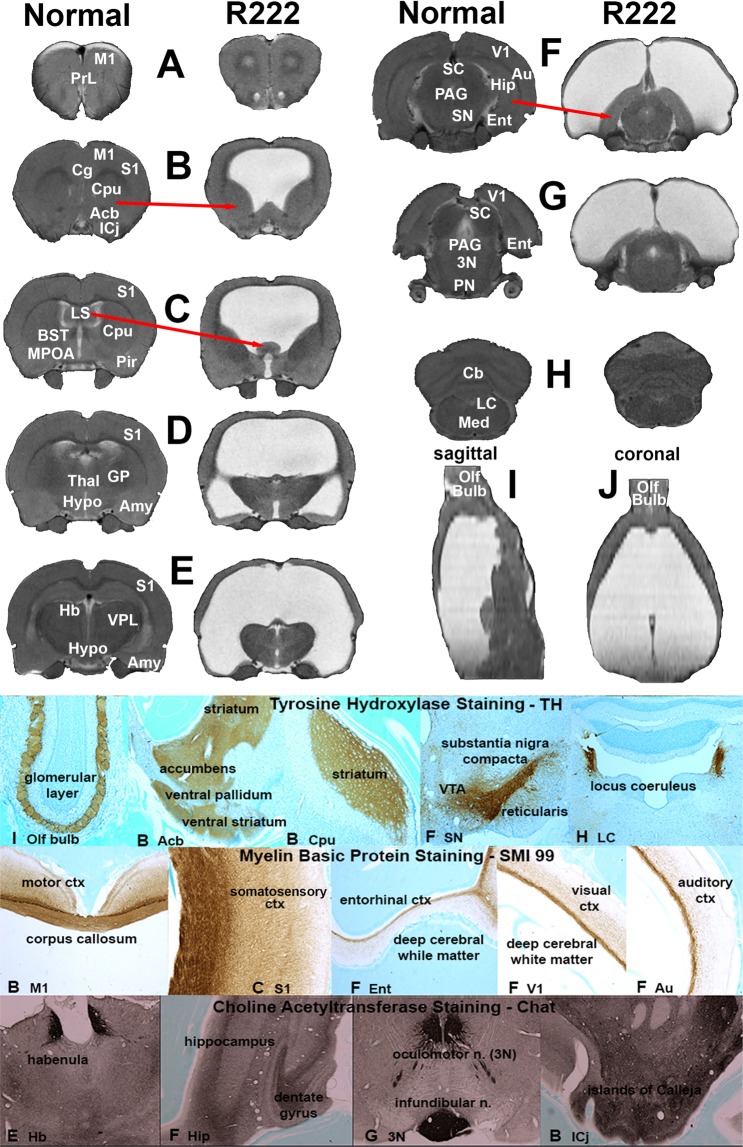
Comparative Neuroanatomical Composite. Shown are serial MR images pairing comparable axial sections between R222 and an age matched control. The images of R222 have been scaled down in size to match that of the control to aid in the comparisons. The photomicrographs at the bottom show immunostaining in different brain areas in R222 for the catecholamines (tyrosine hydroxylase), acetylcholine (choline acetyltransferase) and white matter tracts (myelin basic protein). The lettering on the bottom panel (immunostaining) correspond to the lettering in the top panel (neuroradiography). Abbreviations: M1 primary motor ctx, S1 primary somatosensory ctx, PrL prelimibic ctx, Cpu caudate/putamen, Acb accumbens, ICj Islands of Calleja, Cg cingulate ctx, LS lateral septum, BST bed nucleus stria terminals, MPOA medial preoptic area, Pir piriform ctx, Thal thalamus, GP globus pallidus, Hypo hypothalamus, Amy amygdala, Hb habenula, VPL ventral posterolateral thalamus, SC superior colliculus, PAG periaqueductal gray, SN substantia nigra, Hip hippocampus, V1 primary visual ctx, Au auditory ctx, Ent entorhinal ctx, PN pons, 3N oculomotor n., Cb cerebellum, LC locus coeruleus, Med medulla oblongata, Olf bulb olfactory bulbs.

R222 was a 24 months old *RNaseT2* KO rat. A recently published paper, describes the characterization of the *RNaseT2* KO rat using MRI^11^. These transgenic rats were developed by Horizon Discovery (St Louis MO, USA) in collaboration with Alexion Pharmaceuticals (New Haven CT, USA) to study cystic leukoencephalopathy in response to RNASET2 deficiency^12^. Of the combined six transgenic and six WT rats from a two years old cohort only R222 presented with hydrocephaly. R222’s brain was ca twice the size of the average age-matched controls (Fig. 1). The anatomy (not to scale) compares R222 to an *RNaseT2* KO (control) from the two years old cohort (Fig. 2). It was not possible from these images of R222 to identify the caudate/putamen, amygdala, or hippocampus and the visual, auditory and entorhinal cortices were reduced to a ribbon of tissue (Fig. 2F). While the olfactory bulbs (Fig. 2I,J), prefrontal ctx (Fig. 2A), brainstem and cerebellum (Fig. 2H) were recognizable, the thalamus, hypothalamus and midbrain^13^ were compressed together and pushed caudally toward the floor of the cranium. As many brain areas were unrecognizable, greatly reduced in size or displaced from their original location, they could only be identified by immunostaining for different neurotransmitters. Tyrosine hydroxylase staining revealed the location of dopamine terminals in the reshaped basal ganglia, e.g. striatum (caudate/putamen), accumbens, and ventral striatum (Fig. 2B). The source of these terminals, the midbrain ventral tegmental area and substantia nigra compacta is shown in Fig. 2F. The locus coeruleus, the major source of norepinephrine innervation to the forebrain, remains intact (Fig. 2H). The glomerular layer of the olfactory bulbs had the expected tyrosine hydroxylase staining from intrinsic dopaminergic neurons^14^ but the granular and plexiform layers are devoid of staining (Fig. 2I) suggesting the norepinephrine innervation from the locus coeruleus^15^ is disrupted in R222. Choline acetyltransferase (Chat) staining revealed the location of acetylcholine (Ach) in the hippocampus (Fig. 2F). The hippocampal complex is dramatically reshaped and relocated as shown at level F in Fig. 2 with Nissl stain. Chat staining in the habenula helped to identify the thalamus (Fig. 2E), while staining in the oculomotor n. and infundibular nuclei confirmed the location of the pons (Fig. 2G). The islands of Calleja, the major source of Ach innervation to the cortex, is shown at level B in Fig. 2. Staining for myelin basic protein helped identify the major myelinated tracts, and the reorganization of the cerebrum particularly in the areas of the visual, auditory and entorhinal cortices (Fig. 2F).

### Behavior

**Figure 3:**
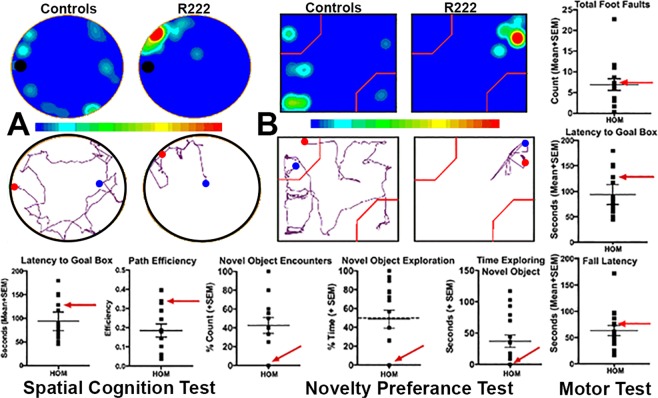
Cognitive and Motor Behavior. (**A**) Movement heat map (top) and track trace (below) of intact rat performance (left) vs R222 (right) on Day 4 trial 3 of the acquisition phase of the Barnes maze. When compared to the intact cohort (n = 13), quantitatively (Bottom), the goal box latency and path efficiency for R222 were within the normal ranges. Qualitatively, the heat and trace maps show R222 explored the maze in an atypical manner and spent most of the time resting in one position. (**B**) Heatmap (top) and track trace (below), as well as novel object encounters count and exploration time data (bottom) for novel object preference performance for a typical, intact rat (left) vs R222 (right). Unlike all intact rats, R222 did not explore the novel or familiar objects and instead huddled in the corner suggesting reduced goal-oriented behavior. This lack of behavior is not likely a function of diminished motor activity since R222 had an average level of performance on the balance beam (top and middle) and the rota-rod (bottom). Novel object preference data for R222 are depicted by red arrows.

The behavior contrasts the differences between R222 to the combined 11 transgenic and WT rats (Fig. 3). Indices of spatial memory and learning across the reported Barnes maze parameters (A) show that R222 (as indicated by the red arrow in the figures) was within the normal range of behavior, compared to the age matched cohort. Pictured in the top left of the figure are heat maps (top) and movement traces for both a typical member of the cohort (left) and R222 (right) on day 4 of the last acquisition trial. Conversely, NOP testing showed that, despite the ability to function within the norm during behavior testing on the Barnes maze, R222 showed little movement and/or interest in the novel object; this could be an indicator of increased anxiety^16^, as rodents typically explore (novel) objects in their environment. In the case of R222, there was no movement approaching either of the objects, with the rat spending the majority of the time huddled in the corner of the arena, a behavior typical to anxiety states. The novel object is demarked by top left area of each NOP heat map, outlined in red. As shown, R222 primarily huddled in the top right portion of the testing box and did not investigate either object (see b graphs). This is in contrast to a typical cohort member (shown on the left side of the block of four heat maps and line traces for NOP) that showed both movement (as indicated by the movement trace in the bottom left of the block), and interest in both objects, as indicated by the heat traces near both the novel (top right) and familiar (bottom right) objects. Motor behavior would appear to be unaffected as R222 performed within the range of the of cohort in the balance beam.

### BOLD imaging

With the discovery of R222 we set out to interrogate the brain using BOLD (blood-oxygen level-dependent) fMRI to map the response of the brain to odor, touch and light stimulation. These BOLD studies would normally be done with R222 fully awake^17^. However, we decided to avoid the stress of several days of acclimation and continued the imaging studies with R222 under light 1% isoflurane anesthesia. While anesthesia significantly dampens the changes in BOLD signal and reduces the global pattern of activation it does provide clear stimulus responses in rats to odor^18^, touch^19^ and light^20^ without motion artifact.

**Figure 4:**
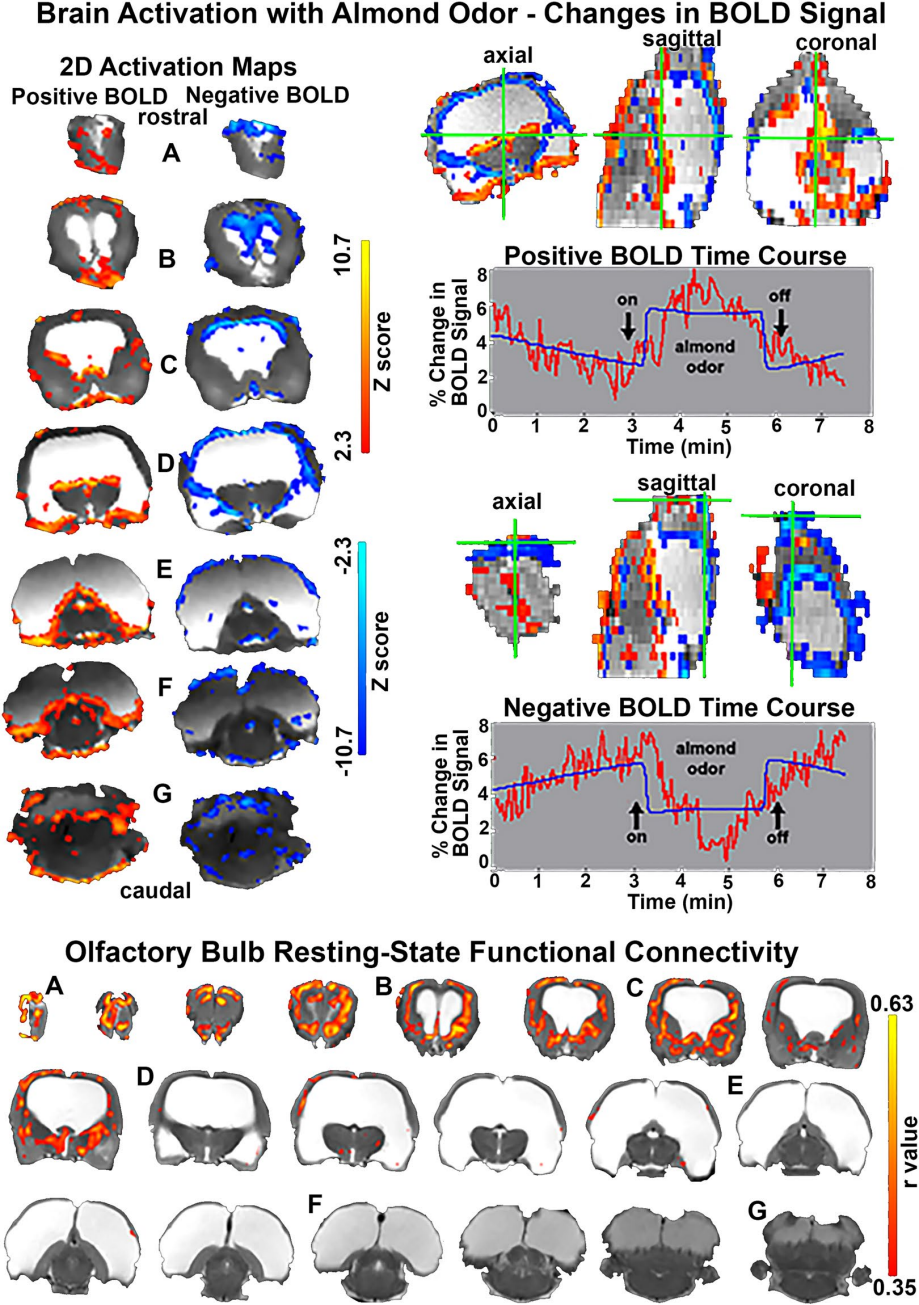
R222 fMRI and resting state connectivity profiles. Top images to the left show maps of positive (red/yellow) and negative (blue) BOLD signal change in response to the odor of almond. The images and time course plots to the right show voxels identified in the green crosshairs as increasing (positive BOLD) or decreasing (negative BOLD) in signal in phase with the presentation of the odor stimulus. Bottom images show brain areas with significant functional coupling to the olfactory bulbs.

A composite of BOLD signal changes in response to the smell of almond and under resting conditions without odor stimulation is shown in Fig. 4. R222 has an intact olfactory bulb, anterior olfactory, olfactory tubercles and piriform ctx. Indeed, the forebrain with prelimbic, infralimbic, medial and lateral orbital cortices and association ctx appear intact as shown in Fig. 1. Hence it was not surprising that R222 shows a pattern of activation that includes these areas that comprise the primary olfactory system. However, the brain activation to almond odor includes both robust positive and negative changes in BOLD signal across much of the brain including the brainstem and cerebellum. Negative BOLD is primarily confined to the thin cortical mantel (Fig. 4D–F) olfactory bulbs (Fig. 4A), forebrain (Fig. 4B,C) and brainstem/cerebellum (Fig. 4G). The positive BOLD was localized to the forebrain olfactory system, the entorhinal ctx (Fig. 4E,F), hippocampus (Fig. 4E,F) and brainstem/cerebellum (Fig. 4F,G). An example of the time course for change in positive and negative BOLD signal with a 3 min presentation of almond odors is provided. When the olfactory bulb is seeded for resting state connectivity the areas of positive correlation cluster around the primary olfactory system of the forebrain (Fig. 4A,B) and caudally to include the hypothalamus and amygdala (Fig. 4C). Interestingly the connectivity does not extend to the hippocampus, pons, and brainstem - all areas activated the smell of almond.

**Figure 5:**
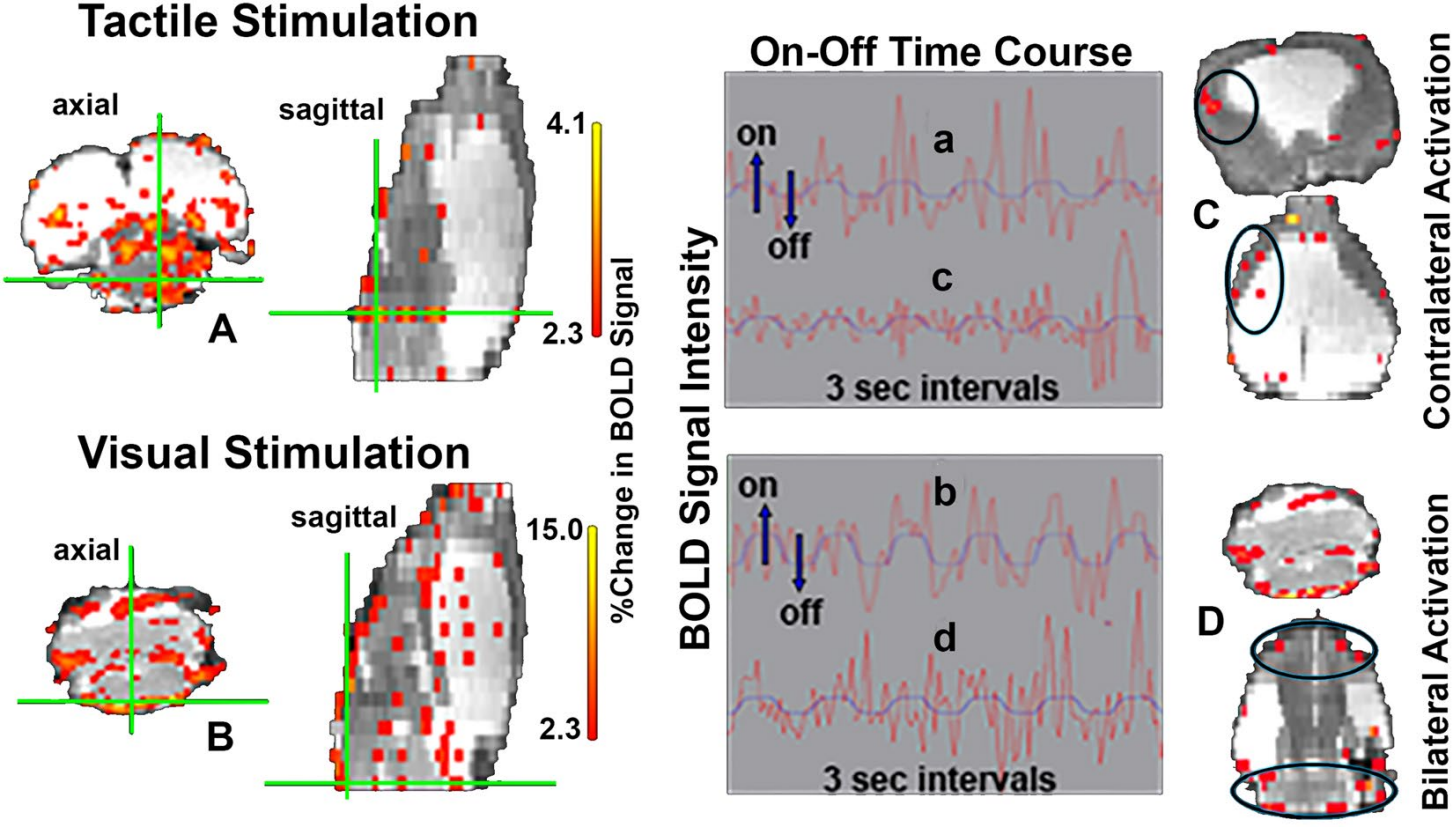
BOLD fMRI in response to tactile and visual stimulation. The top row shows activation maps (**A,C**) and time course plots for voxels in the green crosshairs (a) or circled in black (c) in response to tactile stimulation (shock in right hindpaw). The bottom row shows activation maps (**B,D**) and time course plots for voxels in the green crosshairs (b) or circled in black (d) in response to a flashing white light. The time course plots show the voxels are in phase with the stimuli.

The upper panels (A & C) of Fig. 5 show the change in BOLD signal in response to foot shock (3 mA) delivered to the right hind paw at 3 sec intervals. Panel A shows axial and sagittal sections with positive BOLD signal (red/yellow). The green cross-hairs mark a voxel whose pattern of activation is shown in the time course graph *a*. The axial and coronal sections to the far right (C) demarked in blue depict activation in the somatosensory ctx, contralateral to the foot shock. The pattern of activation to repetitive stimulation is shown in the time course graph *c*.

The bottom panels (B & D) of Fig. 5 show the change in BOLD signal in response to a flash of white light given at 3 sec intervals. Panel B shows axial and sagittal sections with positive BOLD signal. The green cross-hairs mark a voxel whose pattern of activation is shown in the time course graph *b*. The axial and coronal sections in the bottom right (D) demarked in blue show bilateral activation in the frontal ctx and brainstem/cerebellum. The pattern of activation to repetitive stimulation is shown in the time course graph *d*.

## Discussion

Survival requires sensing the environment, processing the information, and responding appropriately. There is no mortal threat or competition to survive for a rat maintained in a cage for two years under controlled environmental conditions with food and water ad libitum. R222 is a study of the fundamental neurobiological and behavioral processes that sustain a resource adequate, safe, ambulatory life. The severity of the hydrocephalus in R222 in the face of normal body weight and growth, normal motor behavior and spatial memory, and evoked activity to smells, tactile stimulation and vision would suggest neuroadaptation to a life-long abnormality. This rare case can be viewed as one of nature’s miracles providing the unique opportunity to examine the brain’s capacity for neuroplasticity and reorganization necessary for survival.

The organization of the cortical mantle represents the brain area most affected by the severe hydrocephalus. The extreme rostral to caudal progressive thinning of the cortical mantle has been reported in genetic and experimental models of hydrocephaly in rats^21,22^. In neonatal rat models, the unfused cranium expands to accommodate the increased ventricular volume. As a result, the cortical mantle stretches covering a larger surface area but retains its original volume. At first glance, one assumes there is a significant loss of cortical volume based on the tissue representing the cortical mantle in the hindbrain of R222. However, the total cortical volume of R222 is only 7% less than age matched controls (see Fig. 1). Because of the enlarged cranium, we might assume R222 was a neonate when afflicted with hydrocephaly. The organization of prefrontal ctx remains intact (Fig. 2 section B). This is note-worthy because of its involvement in many cognitive processes^23^. The primary and secondary motor cortices in the forebrain necessary for volitional movement appear intact. The somatosensory, auditory, and visual cortices are dramatically reduced in size or essentially absent as towards caudal regions. Nonetheless, R222 still responds to foot-shock, startles to a loud sound, and responds to a flashing light. Electrical stimulation of the right hindpaw appears to cause an increase in BOLD signal in what would be the area of the contralateral primary somatosensory ctx. The thalamus is compressed toward the basal hindbrain so the normal connections from ventral posteriolateral thalamus to somatosensory ctx are not obvious. While the visual ctx is absent of an evoked activity and appears reduced to myelinated fibers in passage, the forebrain and hindbrain show bilateral activation to a light stimulus. Given the noise produced in the scanner during the imaging session we were not able to test for hearing. Instead we put R222 in a testing arena and observed a startle response to a loud noise. By these accounts the motor and somatosensory cortices maintain their prescribed ontological neuroanatomical organization and function. However, this would not be the case for the senses of vision and hearing.

In the cortex necessary? There have been numerous studies across a variety of mammals looking at the developmental consequences of radical decortication in neonates^24–30^. While there are minor deficits particularly in some motor patterns and motor coordination, the decorticate animal can eat, drink, sleep and grow to normal size. They respond to visual and auditory stimuli. They display normal species-specific social, maternal, aggressive and sexual behaviors. They mate and reproduce. Nature again has provided science with an extreme form of decortication in humans – hydranencephaly, a rare, inherited disorder where by babies are born without cerebral hemispheres. There is no treatment, yet incredibly, with the proper care and stabilization these individuals can live for years^31^, not in a vegetative state and are responsive to their surroundings.

Did R222 survive on an olfactory system, cerebellum and brainstem? R222 has an intact olfactory bulb, anterior olfactory, olfactory tubercles and piriform ctx. Indeed, the forebrain with prelimbic, infralimbic, medial and lateral orbital cortices and association ctx appear intact. BOLD imaging in response to almond odor showed robust positive and negative BOLD. Negative BOLD is primarily confined to the thin cortical mantel while positive localized to the basal hindbrain. Positive BOLD appears in the olfactory bulbs and forebrain in addition to the brainstem/cerebellum. However, rsFC shows the correlations in BOLD signal were limited to the olfactory bulbs and forebrain. The extension of communication across the long axis of the brain between two seemingly disparate regions is not unprecedented. The cerebellum is consistently activated in human imaging studies that use an odor stimulus^32^. Both the olfactory bulb and cerebellum have numerous polysynaptic connections to much of the brain^33^. While the pathway from the olfactory bulbs to cerebellum has not yet been defined, their primary connection may be through the 5^th^ cranial nerve as the perception of many odors involves the interaction of olfactory bulbs and the trigeminal system^34^. Data based on changing odor intensity suggest that the intranasal trigeminal system may be responsible for odor-induced activation of the cerebellum^35^. The primary sensory n. of the trigeminal nerve has reciprocal connections with the cerebellum^36^.

The cerebellum has reciprocal interactions with much of the brain^36^. Excitatory outputs from the cerebellar nuclei impact the motor and somatosensory cortices, thalamus, hypothalamus, amygdala, basal ganglia, and hippocampus. The connectivity to these brain regions is not unexpected given the growing literature on the cerebellum’s involvement in emotion and cognition, and its reciprocal connections to these areas^37,38^. Perhaps the cerebellum compensates for the diminished volumes and presumptive functions of the amygdala, somatosensory and limbic cortices and hippocampal complex.

Studies on the evolution of encephalization in Mammalia point to the importance of the olfactory system and cerebellum. Rowe and colleagues report the evolutionary events (i.e. pulses) leading to the origin and culmination of the mammalian brain was driven by expansion of the olfactory bulbs and olfactory ctx^39^. Barton and Venditti proposed cerebellar specialization was a far more important component of human brain evolution than previously thought, as evidenced by a more rapid increase in size compared to the neocortex^40^. These studies repudiate the common notion that encephalization and brain enlargement was driven by neocortical development. Instead, they point to the critical role played by the positioning of the olfactory system and cerebellum at opposite ends of the neural axis.

What is the bare minimum? An intact brainstem is essential for all vegetative functions e.g. autonomic regulation of respiration, heart rate, blood pressure. A hypothalamus and pituitary gland are necessary for ingestive, social and sexual behaviors, homeostasis, endocrinology, salt and water balance, body temperature, and circadian rhythms. Motor ctx, basal ganglia, and thalamus are necessary for goal directed movement. The hippocampus is needed for memory. The primary sensory modalities, smell, visual, hearing and touch are needed to sense the external world. In the case of rodents and many other species, olfaction is essential^41^. Between the behavioral testing, MRI, and post mortem histology, it was possible to observe many of these functions, and confirm their location and organization in R222. The transformation of cortical mantle was the most dramatic change in R222 as compared to age matched controls, particularly the visual and auditory cortices. How did R222 see and hear? In a review on the functional organization of the vertebrate brain, Merker makes a compelling argument for the upper brainstem e.g., thalamus, hypothalamus, midbrain DA system, superior/inferior colliculi, and pons for the integration of sensory information needed for goal directed behavior in real time^42^. All these areas have access to somatosensory, visual and auditory information independent of the cortex. Long before encephalization there was centralization of function in the upper brainstem for sensory integration, learning, memory, motivation, and organization and expression of complex behaviors. This centralization of life functions in subcortical areas would include the olfactory bulbs and cerebellum as noted above. R222 lived a long “normal” life by defaulting to brain organization that has sustained and propagated vertebrate life since inception.

Could *RNAse T2* mutation be the cause of the R222’s hydrocephaly? Ribonuclease T2 is a protein that breaks down RNA. The absence of this protein at birth leads to RNAse T2-deficient leukoencephalopathy, an abnormality in brain white matter causing a host of neurological problems. Hydrocephaly is not a hallmark of RNAse T2-deficient leukoencephalopathy; instead, afflicted individuals often present with a small head and brain size (https://ghr.nlm.nih.gov/condition/rnase-t2-deficient-leukoencephalopathy). If the *RNAse T2* mutation is not the cause of the hydrocephaly mutation is it possible the absence of RNAseT2 is responsible for the longevity of R222?

## Supplementary information

Supplementary methods
