# Supplementary material for: Life without a brain: Neuroradiological and behavioral evidence of neuroplasticity necessary to sustain brain function in the face of severe hydrocephalus
Source: Sci Rep. 2019 Nov 11;9:16479. doi: 10.1038/s41598-019-53042-3 (PMC6848215; doi:10.1038/s41598-019-53042-3)
Supplement: Supplementary file 1 — Supplementary methods [file 41598_2019_53042_MOESM1_ESM.pdf]

Life without a brain: Neuroradiological and behavioral evidence of neuroplasticity necessary to sustain brain function in the face of severe hydrocephalus

C.F. Ferris, X. Cai1, J. Qiao, B. Switzer, J Baun, T. Morrison, S. Iriah1, D. Madularu, K.W. Sinkevicius, P. Kulkarni

Supplementary Material

Primary Antibody:	NeuN IHC
Source:	Abcam
Catalog #:	ab104225
Host:	Rabbit
Dilution: 1:	3K
Chromagen:	NiDAB
Color:	Black
Secondary Antibody:	Anti-Rabbit Biotinylated
Source:	Vector
Catalog #:	BA-1000
Host:	Goat
Dilution: 1:	1,000

Primary Antibody:	Tyrosine Hydroxylase IHC
Source:	Pel Freez
Catalog #:	P40101
Host:	Rabbit
Dilution: 1:	18K
Chromagen:	DAB
Color:	Brown
Secondary Antibody:	Anti-Rabbit
Source:	Vector
Catalog #:	BA-1000
Host:	Goat
Dilution: 1:	1,000

Primary Antibody:	Serotonin 5HT
Source:	Immunostar
Catalog #:	20080
Host:	Rabbit
Dilution: 1:	15K
Chromagen:	DAB
Color:	Brown
Secondary Antibody:	Anti-Rabbit
Source:	Vector

Catalog #: BA-1000
Host: Goat
Dilution: 1: 1,000

Primary Antibody: ChAT IHC
Source: Millipore
Catalog #: ab144P
Host: Goat
Dilution: 1: 750
Chromagen: Ni-DAB
Color: Black
Secondary Antibody: Anti-Goat
Source: Vector
Catalog #: BA-5000
Host: Rabbit
Dilution: 1: 238

Primary Antibody: MBP (SMI-99) IHC
Source: BioLegend
Catalog #: 808402
Host: Mouse
Dilution: 1: 1500
Chromagen: DAB
Color: Brown
Secondary Antibody: Anti-Mouse
Source: Vector
Catalog #: BA-2001
Host: Horse
Dilution: 1: 1,000
